# A rare case of chicken wishbone in Meckel’s diverticulum causing partial perforation and diverticulitis

**DOI:** 10.1093/jscr/rjae519

**Published:** 2024-08-25

**Authors:** Irada Mamukadze, Sean Park, Olivia Flessland, Libby Moberg, Amy Jentz

**Affiliations:** Department of General Surgery, University of Michigan Health-Sparrow, 1215 E. Michigan Avenue, Lansing, MI 48912, United States; Department of General Surgery, University of Michigan Health-Sparrow, 1215 E. Michigan Avenue, Lansing, MI 48912, United States; Department of General Surgery, University of Michigan Health-Sparrow, 1215 E. Michigan Avenue, Lansing, MI 48912, United States; Department of General Surgery, University of Michigan Health-Sparrow, 1215 E. Michigan Avenue, Lansing, MI 48912, United States; Department of General Surgery, University of Michigan Health-Sparrow, 1215 E. Michigan Avenue, Lansing, MI 48912, United States

**Keywords:** perforated Meckel’s diverticulum, foreign body in Meckel’s diverticulum, bowel obstruction

## Abstract

Meckel’s diverticulum (MD), a rare congenital abnormality, can lead to issues like diverticulitis and bleeding. Foreign bodies in MD are even rarer, causing vague symptoms and perforation, requiring urgent surgery. This case report highlights a patient with a foreign body in MD, focusing on clinical presentation and management. A 55-year-old male presented with abdominal pain, nausea, and vomiting. Computed tomography scan revealed a foreign body perforating the small bowel. Exploratory laparotomy found a partially perforated MD with a foreign body. Diverticulectomy was performed, and the patient recovered, discharged the next day. Foreign bodies in MD are exceedingly rare and can cause inflammation, infection, and perforation, mimicking appendicitis. Diagnosis is challenging due to nonspecific symptoms, with imaging and clinical evaluation crucial. Surgical intervention, like diverticulectomy, is primary. Early diagnosis and prompt surgery are critical in managing MD complicated by foreign bodies, ensuring favorable outcomes. This report underscores symptom recognition and effective management strategies.

## Introduction

Meckel’s diverticulum (MD) is a rare congenital abnormality, named after Meckel Friedrich in 1809 [1]. Phillipos *et al.* [2] asserted that MD congenital anomalies could trigger diverticulitis and gastrointestinal bleeding, strengthening its clinical significance. The presence of a foreign body in MD is even less common and can lead to vague abdominal symptoms and perforation, potentially becoming a medical emergency that requires surgical intervention. Consequently, it is imperative to have case reports that can describe patient symptoms and presentations, enabling the recognition of warning signs and accurate diagnosis and management. Therefore, we are pleased to present a case report of a patient who experienced abdominal pain and was subsequently found to have a foreign body lodged in MD.

## Case presentation

**Figure 1:**
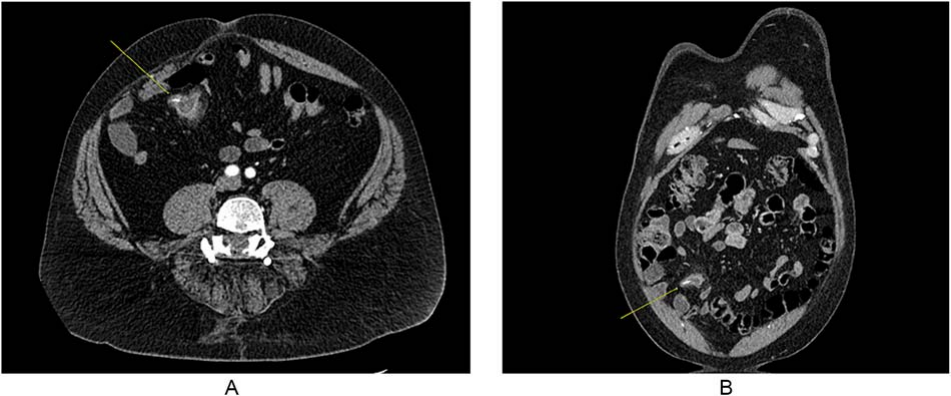
CT imaging showing foreign body in MD, with some inflammation and fat stranding around it. (A) Coronal imaging and (B) cross-sectional.

**Figure 2:**
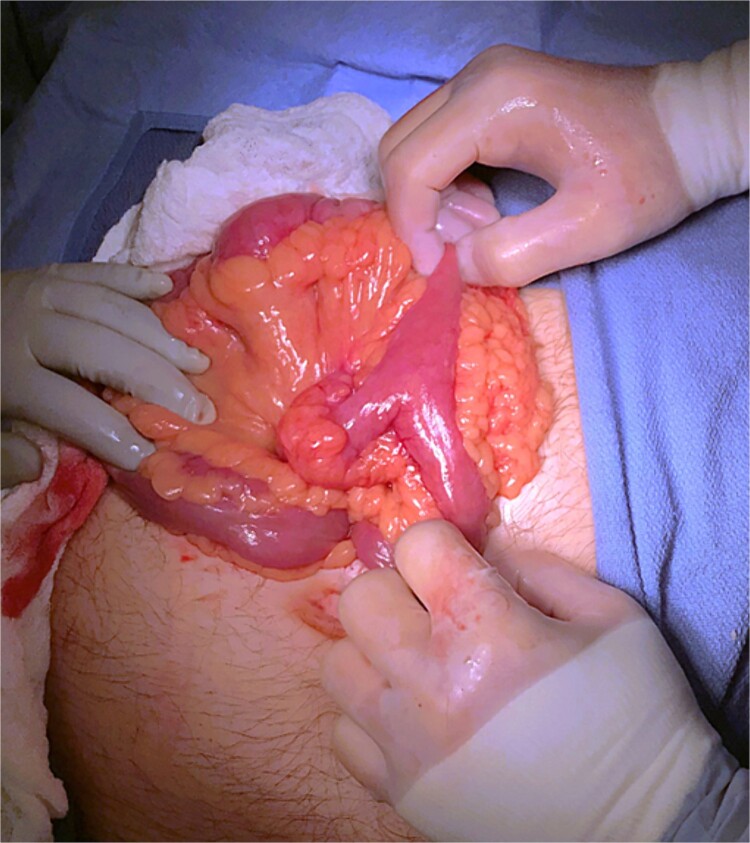
Intraoperative image of MD.

**Figure 3:**
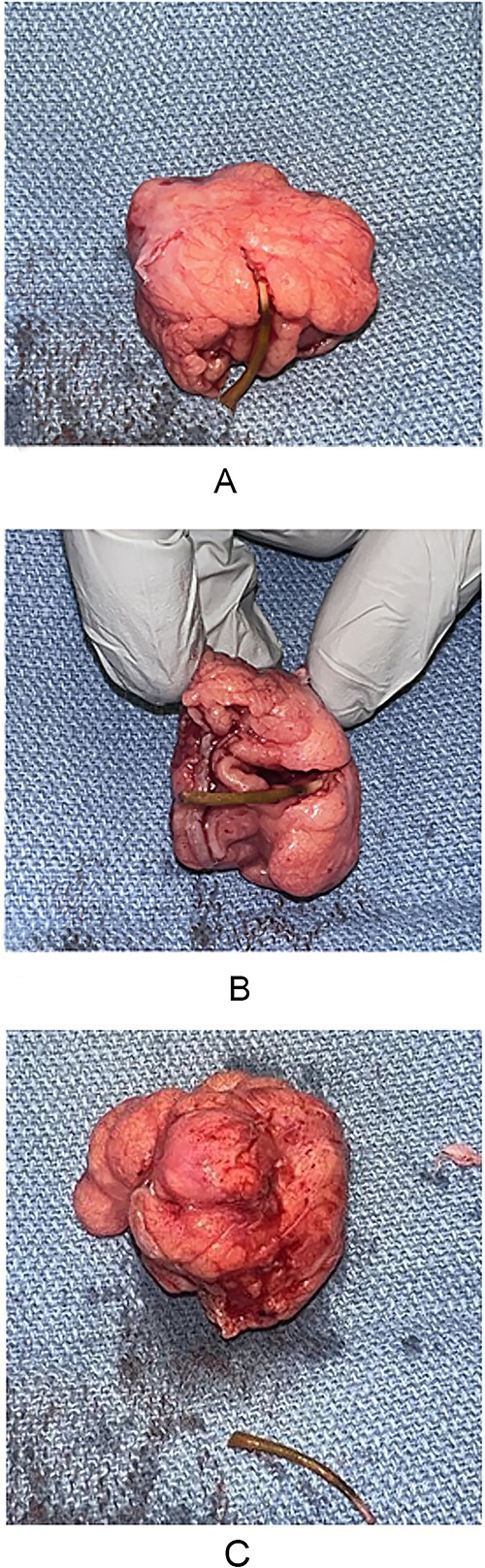
(A–C) MD specimen with foreign body.

A 55-year-old male presented to the Emergency Department with right upper quadrant abdominal pain, nausea, and vomiting. A computed tomography (CT) scan (Fig. 1) revealed a linear foreign body that appeared to perforate the small bowel on the right side, accompanied by inflammation. No free air or fluid was observed. He did not recall ingesting anything containing bones. The decision was made to take the patient to the operating room for exploratory laparotomy, during which it was discovered that the patient had a partially perforated MD with inflammation and a foreign body inside (Figs 2 and 3). Inflammation and perforation were located at the distal end of the MD with a healthy base. Therefore, diverticulectomy was performed without any significant compromise or stricture of the ileum. The abdomen was then irrigated and closed, and the patient was transferred to the post-anesthesia care unit without any complications. The patient’s postoperative course was uncomplicated and he was discharged on postoperative Day 1.

## Discussion

MD is a rare congenital anomaly that can cause significant issues like diverticulitis and bleeding. The presence of a foreign body in MD is even rarer, potentially leading to vague symptoms, perforation, and the need for urgent surgery.

Common foreign objects in MD include fish bones, dental appliances, and small toys [3]. These can cause irritation, inflammation, and infection, often mimicking acute appendicitis with symptoms like abdominal pain, nausea, vomiting, and fever [4, 5]. Diagnosing MD is challenging due to nonspecific symptoms, requiring imaging and clinical evaluation [6].

Surgical intervention, such as diverticulectomy or bowel resection, is the primary treatment for foreign body-related MD. Early diagnosis and prompt surgery are crucial to avoid severe complications like abscess formation, perforation, and sepsis [7]. This case report underscores the importance of recognizing symptoms and provides insights into effective management strategies.

Most MD patients remain asymptomatic, but complications can arise from obstruction or infection, often resembling appendicitis [4]. Imaging modalities like CT scans and endoscopic procedures are valuable for diagnosis [3, 8]. Laparoscopic surgery is often preferred due to shorter recovery times.

Timely surgical intervention and accurate diagnosis are vital for favorable outcomes in MD cases with foreign bodies. More case reports are needed to detail symptom recognition and management of this rare condition [9–12].

## Conclusion

MD is an infrequent medical condition, and the presence of foreign bodies within it is rare. Consequently, case reports that shed light on patients presenting with foreign bodies are vital for facilitating symptom recognition and guiding proper management. This case report underscores the importance of early diagnosis and intervention in cases of MD complicated by foreign body dislodgement to ensure the best possible patient outcomes.
